# Dysfunctional Attitudes, Sociotropy–Autonomy, and Intimate Partner Violence Victimization in Emerging Adulthood

**DOI:** 10.3390/ijerph20126164

**Published:** 2023-06-18

**Authors:** Chloé Cherrier, Robert Courtois, Emmanuel Rusch, Catherine Potard

**Affiliations:** 1 UR 1901 QualiPsy, Department of Psychology, University of Tours, 37041 Tours, France; robert.courtois@univ-tours.fr; 2 EA 7505 EES, Department of Public Health, University of Tours, 37044 Tours, France; emmanuel.rusch@univ-tours.fr; 3 UR 4638 LPPL, Department of Psychology, University of Angers, 49045 Angers, France; catherine.potard@univ-angers.fr

**Keywords:** intimate partner violence, victimization, sociotropy–autonomy, dysfunctional attitudes, emerging adulthood

## Abstract

Understanding the dynamics and vulnerability factors involved in intimate partner violence (IPV) victimization among emerging adults is important in order to better prevent it from happening. The current study aimed to investigate the relationships among dysfunctional attitudes, sociotropy–autonomy, and types of IPV victimization (i.e., psychological, physical, and sexual) and severity (i.e., minor or severe) in emerging adulthood. Through an online survey, 929 emerging adults (84.6% women, mean age = 23.61) completed self-report questionnaires related to variables explored. When checking for childhood abuse, dysfunctional attitudes, sociotropy, and autonomy were related to IPV victimization for at least one type of violence and one scale of severity. The regression models show that independence from others and importance given to others are related to greater severe and minor physical violence, respectively. Attraction to loneliness seemed related to lesser minor psychological violence, whilst valorization of freedom of movement and action were related to greater minor sexual violence. The capacity to oppose others seemed related to greater severe sexual violence. These different cognitive and social characteristics may be associated with poorer social skills, thus making emerging adults more vulnerable to IPV victimization. The preventive and clinical implications are discussed.

## 1. Introduction

Having romantic relationships free of violence is an important precondition for wellbeing during emerging adulthood. Emerging adulthood is a developmental period between late adolescence and adulthood (18–30 years) where a field of possibilities is explored, particularly in romantic relationships [1]. However, emerging adults are particularly affected by intimate partner violence (IPV) since it has been estimated that 45.2% of young women and 40.8% of young men first experience some form of IPV between the ages of 18 and 24 years old [2]. IPV is defined as “behavior within an intimate relationship that causes physical, sexual, or psychological harm, including acts of physical aggression, sexual coercion, psychological abuse, and controlling behaviors” [3] (p. 11). Young men and women seem equally affected by these different forms of IPV [4,5], except for sexual violence, whereby women are the main victims and men the main perpetrators [6,7]. IPV victimization is associated with many negative consequences for wellbeing, including depression, stress, physical injury, alcohol use, and socioeconomic issues, as well as the risk of revictimization [3,8,9]. In addition, being a victim of one form of IPV is often associated with the risk of being a victim of another form of IPV, a phenomenon known as polyvictimization [6]. Identifying intrinsic vulnerability factors is important in order to prevent IPV [10]. The purpose of this study is to explore the links among dysfunctional attitudes, sociotropy–autonomy, and IPV victimization in emerging adulthood.

The vulnerability factors most involved in IPV are often related to the family environment (e.g., childhood exposure to violence, parental attachment, and witnessing interparental partner violence) [8,11,12]. An insecure or violent family environment can impact the development of the individual and, therefore, these cognitive schemas. These aforementioned early maladaptive schemas are dysfunctional internal working models [13]. Acceptance of violence becomes normalized and is transmitted from generation to generation following social learning theory, making the individual more vulnerable to IPV later in life [14,15]. Beck’s cognitive theory (1983) postulated that maladaptive beliefs formed in childhood will be retained in adulthood and will lead to dysfunctional attitudes when negative or stressful events occur [16]. Dysfunctional beliefs promote information processing errors, and produce negative thoughts about the self, others, the world, and the future. Once established, these patterns serve as a guide to behavior, influencing how new information is assimilated, and can lead to dysfunctional attitudes. The latter, often studied as a component of depressive self-schemas, are defined as negative, excessive, and inflexible, with “if–then” statements concerning the self or others [17]. Moreover, it has been well established that antecedents of childhood or sexual abuse contribute to the development of more dysfunctional attitudes [18,19,20]. These dysfunctional cognitive schemas can, therefore, negatively impact conflict resolution skills in romantic relationships and be associated with IPV. A study by Kaygusuz (2013) [21] showed in the student population that having dysfunctional beliefs in romantic relationships was associated with psychological and physical victimization, as well as poorer problem-solving skills. Beyond these beliefs related to acceptance of violence in romantic relationships, relatively little empirical research has been conducted on the topic of cognitive characteristics of IPV victims. Given the importance of these beliefs, it is essential to identify distorted thoughts not only among perpetrators but also among the victims. The meta-analysis of Pilkington et al. (2021) [22], evaluating nine studies, showed that the need for reassurance in love, the need for security, and doubting one’s ability to assume one’s responsibilities or to succeed in life were all associated with vulnerability in the face of IPV victimization.

One might also hypothesize that having dysfunctional beliefs about oneself and others could have an impact on social skills and one’s relationship with others. For example, in a study on women with an addicted husband on the verge of divorce, it was found that women who had dysfunctional attitudes showed poorer communication skills [23]. Bartholomew (1990) [24] postulated that having negative schemas of the self can be externalized into dependency, (i.e., seeking approval from others in order to maintain positive self-esteem), while negative schemas of others can be externalized as avoidance or autonomy, to avoid closeness and to protect against disappointment. This may refer to the sociotropic or autonomous personality styles developed by Beck [25], and it has been found to be associated with dysfunctional attitudes [26,27]. Sociotropy represents an extreme form of sociability and is characterized by a state of social dependence. A sociotropic person will tend to seek close emotional relationships and avoid experiences of loss, rejection, or abandonment as much as possible [16]. In contrast, autonomy is characterized by the search for more distant and emotionally detached relationships. A highly self-reliant person emphasizes individuality and personal accomplishments [28,29,30]. It appears that there are gender differences in these personality traits. Women have a significantly higher level of sociotropy than men, while, for autonomy, no difference between the sexes was found [16,30].

Sociotropy–autonomy is often studied in psychopathological conditions, e.g., depression [29,31], suicide, or eating disorders in clinical and general samples [30,32]. Concerning interpersonal context, Sato et al. found that highly sociotropic individuals may exhibit hostile behaviors such as wanting revenge, dominating, or being intrusive, particularly within close relationships [29,33]. Autonomous people tend to withdraw from other relatives and prefer solitude. Moreover, in a population of emerging adults, Sibley and Overall (2008) [34] showed that a high level of autonomy was associated with low sociability regarding interactions with romantic partners. Thus, sociotropy–autonomy has been shown to have an effect on interactions between couples [35]. Conversely, a high level of sociotropy was associated with increased sociability in social interactions. It can also be considered that these dimensions could paint a picture of one’s relationship to others and, thus, give an overview of social skills, such as being comfortable in interpersonal relationships or knowing how to communicate effectively. A Turkish study on a social skills development program among students showed that the post-test scores of the study group on the sociotropy subscale were lower than their pre-test results, suggesting that these dimensions could serve as an indicator to measure social skills [36]. Within the research to date, only one study examined links between sociotropy–autonomy and IPV, and that was only for psychological violence [37]. Their results showed that female victims had a higher level of sociotropy.

In line with previous findings, the present study aims to explore whether dysfunctional attitudes and sociotropy–autonomy are associated with the severity of IPV victimization in its diverse forms (i.e., psychological, physical, and sexual) in emerging adulthood. Since childhood abuse is linked to both of these vulnerability factors and to IPV, it was checked for during the analyses. We predicted that dysfunctional attitudes and sociotropy–autonomy would be associated with greater severity of IPV victimization in its different forms. 

## 2. Materials and Methods

### 2.1. Participants and Procedure

A total of 929 emerging adults in France (*M*_age_ = 23.61 years, SD = 3.36, range = 18–30 years), with 786 women (84.6%) and 143 men (15.4%), participated in the study. They included 430 (46.3%) students, 139 (15%) students in part-time employment, 272 (29.3%) workers, and 88 (9.5%) unemployed. The mean academic level was 15.76 years (SD = 2.51), with the lowest level being equivalent to the seventh grade (7 years of study) and the highest level being equivalent to a doctorate degree (21 years of study). Participants were of middle or high socioeconomic status (89.6%). The majority of participants reported a heterosexual orientation (*n* = 746; 80.4%). A total of 106 participants reported being bisexual (11.4%), 40 identified as gay/lesbian (4.3%), and 36 (3.9%) participants identified as other or were unsure of their sexual orientation. In addition, 757 (81.5%) were in a romantic relationship at the time of the study, and 172 (18.5%) had been in a romantic relationship in the last 12 months. The study design was cross-sectional. A self-administered online questionnaire designed for young adults (aged 18 to 30) was distributed through various networks in France (university, leisure groups, professionals, etc.). Participants signed a consent form guaranteeing their anonymity. The project was approved by a Research Ethics Committee of Tours-Poitiers (2019-03-04).

### 2.2. Instruments

#### 2.2.1. Sociodemographic and Control Variables

Participants completed a demographic information section that included questions on sex assigned at birth, age, education level, and history of childhood abuse (adaptation of the French version of the Childhood Trauma Questionnaire) [38].

#### 2.2.2. Intimate Partner Violence

The French version of the revised Conflict Tactics Scales (CTS2) [39] was used to assess psychological, physical, and sexual victimization (27 items). Participants reported the frequency of each tactic within the past year on a Likert-type scale ranging from 0 (never) to 6 (more than 20 times). Each scale could be evaluated according to the severity of the assault (minor and severe) [40]. Sample items for the psychological violence scale included “my partner insulted me or swore at me” for minor acts (four items) and “my partner destroyed something belonging to me” for severe acts (four items). For the physical violence scale, sample items included “my partner pushed or shoved me” for minor acts (five items) and “my partner choked me” for severe acts (seven items). Lastly, for minor sexual violence, sample items included “my partner insisted on having sex with me when I did not want to (but did not use physical force)” for minor acts (three items) and “my partner used threats to make me have oral or anal sex” for severe acts (four items). For both the physical and sexual violence scales, items categorized as severe violence have a high potential for injury, which is what differentiates them from those found on the minor violence subscales. The subscales were used either continuously or in categories (absence, minor, and severe). In the current study, Cronbach’s alphas ranged from 0.58 to 0.88.

#### 2.2.3. Sociotropy–Autonomy

The French version of the Sociotropy–Autonomy Scale (SAS) [30] was administered to assess sociotropy and autonomy. Sociotropy refers to people’s motivation to engage in positive interactions with others, divided into three subscales: importance given to others (12 items, e.g., “I am concerned that if people knew my faults or weaknesses, they would not like me”), worry about separation and looking for support (11 items, e.g., “I get lonely when I am home by myself at night”), and attention to others (seven items, e.g., “I feel I have to be nice to other people”). Autonomy refers to people’s motivation to maintain and increase their independence, also separated into three subscales: goal achievement and independence (12 items, e.g., “It is important to me to be free and independent”), attraction to loneliness (11 items, e.g., “I feel more comfortable helping others than receiving help”), and valorization of freedom of movement and action (seven items, e.g., “I don’t like people to invade my privacy”). This five-point Likert-type scale has 60 items. High scores indicate that the relevant dimension is effective. For the current study, Cronbach’s alphas ranged from 0.58 to 0.87.

#### 2.2.4. Dysfunctional Attitudes

The Dysfunctional Attitude Scale—Form A (DAS) [41] evaluates the level of dysfunctional attitudes, according to Beck’s cognitive theory. Dysfunctional attitudes assess dysfunctional beliefs related to the self and others. It is based on 29 items and includes four factors; two refer to sociotropy, seeking the esteem of others (16 items, e.g., “It is difficult to be happy unless one is good looking, intelligent…”) and seeking approval of others (three items, e.g., “If others dislike you, you cannot be happy”), and two refer to autonomy, the capacity to oppose others (six items, e.g., “If someone disagrees with me, it probably indicates that they do not like me”), and independence from others (four items, e.g., “If you cannot do something well, there is little point in doing it at all”) [41]. A high score indicates greater cognitive bias. In the current study, Cronbach’s alphas ranged from 0.52 to 0.88. 

### 2.3. Data Analysis

A description of the severity of the three forms (i.e., psychological, physical, and sexual) of IPV victimization was made according to gender. The prevalence corresponds to having experienced at least one act of IPV during the last year, and the frequency scores correspond to the average number of acts of IPV experienced during the last year. Chi-square, Wilcoxon–Mann–Whitney tests, and Spearman (partial) correlations (controlling for antecedent of childhood abuse) were conducted to evaluate associations among severity of IPV victimization, dysfunctional attitudes, sociotropy–autonomy, and sociodemographic variables. Lastly, multinomial logistic regressions were performed to determine the variables most predictive of the severity of IPV (using absence of violence as the control group). Sample size guidelines for multinomial logistic regression indicate a minimum of 10 cases per independent variable [42]. We reported odds ratios (OR) with 95% confidence intervals. Each of the regression models was adjusted for sex, age, education level, and history of childhood abuse. Statistical analyses were conducted using SPSS^®^ version 25, with a significance level retained at 0.05. 

## 3. Results

### 3.1. Description of the Severity of IPV

Taking into account prevalence and frequency, the scores of exposure to IPV in each form (psychological, physical, and sexual) depending on their severity, reported by women and men, are presented in Table 1. The prevalence of having experienced IPV at least once was 64.0% (*n* = 595; *n* = 507 women, *n* = 88 men) for psychological violence, 16.8% (*n* = 156; *n* = 130 women, *n* = 26 men) for physical violence, and 20.5% (*n* = 190; *n* = 176 women, *n* = 14 men) for sexual violence. Psychological violence seemed to be the most frequent form, followed by physical violence and then sexual violence. Only the prevalence and frequency of sexual violence differed according to gender, with more women victims (of minor form and exclusively of severe form; *χ*^2^ = 11.81, *p* < 0.001) than men, and with greater frequency (*t* = −30.54, *p* < 0.001).

Furthermore, associations among different forms of severity of IPV experienced, sociodemographic characteristics (age and education levels), and history of childhood abuse are shown in Table 2. The three forms of IPV were associated with each other (*r* = 0.12 to 0.47, *p* < 0.001). Physical violence had a stronger association with psychological violence than with sexual violence.

### 3.2. Dysfunctional Attitudes, Sociotropy–Autonomy, and Severity of IPV

The different dimensions of dysfunctional attitudes, as well as sociotropy and autonomy, were crossed with the different forms of IPV according to their severity. These results are shown in Table 3. Overall, dimensions of dysfunctional attitudes were positively associated with three forms of IPV (except seeking the esteem of others and seeking the approval of others, which were less associated with the different forms or severity of IPV). For sociotropy, only the dimension importance given to others was positively associated with the three forms of minor IPV (*r* = 0.07 to 0.08, *p* < 0.05). For autonomy, attraction to loneliness was associated only with minor psychological violence (*r* = 0.10, *p* < 0.01), and valorization of freedom of movement and action was associated with psychological (minor and severe; *r* = 0.08 to 0.09, *p* < 0.05), physical (severe; *r* = 0.10, *p* < 0.01), and sexual (minor; *r* = 0.09, *p* < 0.01) violence. It should also be noted that a history of childhood abuse was positively associated with dysfunctional attitudes *(r* = 0.07 to 0.19, *p* < 0.05 to 0.001), as well as the attraction to loneliness and the valorization of freedom of movement and action subdimensions of autonomy (*r* = 0.12 to 0.17, *p* < 0.001), but negatively associated with the worry about separation and the looking for support subdimension of sociotropy (*r* = −0.11, *p* < 0.01). Partial correlations checking for childhood abuse were generally only slightly lower (*r* = 0.07 to 0.09, *p* < 0.05 to 0.01), and the pattern of significance was the same, except for some relationships that were already weakly significant (also displayed in Table 3).

To investigate in greater depth which variables were the most associated with the severity of the three forms of IPV victimization, we carried out multinomial logistic regressions to determine whether dysfunctional attitudes and/or sociotropy–autonomy were associated with increased vulnerability of minor or major IPV (absence of violence group as reference group; see Table 4 for details). Regarding psychological violence, results indicate that attraction to loneliness (autonomy) lessened minor violence (OR = 0.97, *p* < 0.05). On the other hand, the capacity to oppose others (dysfunctional attitude) and the importance given to others (sociotropy) could lead to increased minor (OR = 1.04, *p* < 0.05) and severe (OR = 1.14, *p* < 0.01) physical violence, respectively. The independence from others (dysfunctional attitude) and the valorization of freedom of movement and action (autonomy) could lead to increased minor (OR = 1.06, *p* < 0.05) and severe (OR = 1.14, *p* < 0.05) acts of sexual violence, respectively. Furthermore, a history of childhood abuse was a vulnerability factor for all three forms of IPV (except for major physical violence).

## 4. Discussion

This study examined the association among dysfunctional attitudes, sociotropy–autonomy, and the severity of all forms of IPV victimization (i.e., psychological, physical, and sexual) in emerging adulthood. Our findings showed that psychological violence was the most common form of violence and was concomitant with physical and sexual violence. There was no gender difference for psychological and physical victimization, but more women than men were victims of sexual violence. The prevalence rates and frequency scores obtained, as well as the associations among the different forms of IPV, were consistent with those in the literature, emphasizing the symmetry of IPV for psychological and physical violence, and the asymmetry for sexual violence [7,43] and polyvictimization [6]. Furthermore, antecedents of childhood abuse were significantly associated to the severity of IPV for its three forms [8,11,12] except for major physical violence, probably explained by a statistical weakness (due to the small number).

### 4.1. Dysfunction Attitudes and IPV

Dysfunctional attitudes linked to the dimensions of autonomy (i.e., the capacity to oppose others and independence from others) were more associated with the different forms of IPV and their severity, unlike the dimensions related to sociotropy (i.e., seeking the esteem of others and seeking approval of others). Since childhood abuse was associated with IPV, once the latter was controlled, the associations between dysfunctional attitudes and IPV were diminished. Although few studies exist on the links between dysfunctional attitudes and IPV, the association found with childhood abuse seems to be consistent [18,19,20]. This could partly explain the impact on belief patterns of the self and others, which would make people more vulnerable to IPV victimization. For example, if an emerging adult has a dysfunctional belief such as believing that you have to be beautiful to be happy, then, with a lack of self-confidence, one could consequently be more susceptible to insults from one’s romantic partner regarding appearance.

Consistent with the bivariate statistics, only the dysfunctional attitudes related to autonomy seemed to increase the risk of being a victim of IPV in its severe forms. Moreover, having dysfunctional attitudes linked to independence from others seemed to be a factor of vulnerability for the major forms of physical violence, and the capacity to oppose others seemed to be a factor of vulnerability for the major forms of sexual violence. Having dysfunctional beliefs about the self and others with a desire for autonomy and independence could result in attitudes that are distant or oppositional to others. Social skills, particularly in communication, may be less developed [23]. A young adult may, thus, become more easily isolated and find it more difficult to seek help, possibly explaining a vulnerability to IPV. For example, one of the “opposition to others” ability items is “If someone disagrees with me, that means they don’t like me”. One can imagine that, if a young person adhered to this belief, after a disagreement, they could be more vulnerable in the face of a nonconsensual sexual relationship, by telling themselves that they would “win back” the love of the other by “accepting” sex even if they did not want to. These dysfunctional attitudes related to autonomy reflect a lack of trust in others, with a desire to be different. The results found are similar to those of Pilkington et al. [22], showing that early maladaptive schemas of disconnection and rejection (i.e., lack of safety, stability, and nurturance) are associated with IPV victimization.

### 4.2. Sociotropy–Autonomy and IPV

Having experienced childhood abuse seemed to be positively associated with autonomy and negatively associated with sociotropy. These results seem to coincide with the literature. In their studies among psychiatric patients, Mendelson et al. (2002) found that autonomy was associated more with childhood abuse and less with sociotropy, or only with psychological maltreatment [44]. Wilson and Scarpa (2015) showed that physical or sexual abuse in childhood was associated with a lack of sociability which could explain our negative association [45]. After checking for childhood abuse, we found that the severity of psychological victimization was significantly related positively to autonomy (i.e., attraction to loneliness and valorization of freedom of movement and action). However, these dimensions did not appear to be predictive in the regression analysis for major psychological violence, but we did find the dimension attraction to loneliness as a protective factor against minor psychological violence. Attraction to loneliness, in this study, did not refer to psychological distress which can be viewed as a factor of vulnerability to psychological violence [46], but rather to a search for independence from others. This dimension, which reflects insensitivity and distance from the needs and concerns of others, suggests that this loneliness primarily reflects the absence of social relationships. Solitary individuals may avoid social contact because they feel that other people hold unrealistic expectations of them [47]. Thus, attraction to loneliness can be characterized by a kind of emotional autonomy, with greater ease of distancing in romantic relationships that can leave less room for psychological violence and, thus, can be seen as a protective factor. Nevertheless, attraction to loneliness is often associated with a lack of social skills [48], which can cause this dimension to be ambivalent in the vulnerability/protection of IPV. 

Acts of minor physical violence were found to be slightly associated with the importance given to others (sociotropy), and this dimension also seemed to slightly increase vulnerability to minor physical violence. Sociotropic people have a need for reassurance and maintaining closeness with others, which can make them tolerate physical violence for fear of losing others or because they have an excessive need to please. The few studies present in the literature on the links between sociotropy and violence have mainly shown an association with psychological violence [37,49]. However, since sociotropy is associated with anxious attachment [34] and there is also a reliable amount of literature on these links with the victimization of physical violence [50], we can safely say that these results seem to point in the same direction. Moreover, given that sociotropic individuals may exhibit hostility, anger, suspiciousness, or controlling behaviors in close interpersonal relationships [29,33], one can imagine that this could lead to destructive conflicts and minor physical violence from both partners (due to the bidirectionality of IPV) causing the other partner to withdraw with a defensive attitude, where they could resort to physical violence to restore distance. Lynch et al. (2001) also spoke of a “demand–withdraw” pattern of dealing with conflict [35].

Lastly, minor sexual violence, especially among women, seemed to be more associated with autonomy and increased vulnerability by 1.06, after checking for childhood abuse. Women with an autonomous personality trait may avoid closeness or maintain interpersonal distance because they find intimacy uncomfortable. In this context, the partner who is seeking intimacy may be unsatisfied and inflict minor sexual violence to “restrain” and control their partner. We also know that autonomy is associated with avoidant attachment, which can be found as a factor of vulnerability to the victimization of sexual violence (see Velotti’s literature review in 2008); the results could be consistent [34,51]. In the same fashion as with dysfunctional attitudes (which refer to autonomy), a self-sufficient person may have fewer social skills, not dare to seek help, and think that they have to solve problems alone.

### 4.3. Limitations and Future Research

Several limitations in the current study should be noted. Firstly, the current study was cross-sectional and was conducted online with only a sample of emerging adults. Furthermore, the over-representation of women did not allow comparisons to be made between men and women, whereas sociotropy could differ according to gender [16,30]. The racial difference was not indicated either, given that this is not common practice in France and requires an important ethical framework. Secondly, some Cronbach’s alphas for IPV, dysfunctional attitudes, and sociotropy–autonomy were relatively low, possibly affecting the strength of associations. Thirdly, retrospective reports are not ideal in that they do not permit tests of causal relations between childhood experiences and dysfunctional attitudes, sociotropy–autonomy, or IPV. Fourthly, some IPV severity groups, including major sexual violence or physical violence, were small in size. Future studies should be conducted with larger samples and a longitudinal design to better represent all types of IPV and show changes over time and/or dyadic interaction. Moreover, this would demonstrate whether dysfunctional attitudes and sociotropy–autonomy predict IPV victimization on the basis of its severity, or whether it is being a victim of IPV that fuels dysfunctional attitudes and sociotropy–autonomy. Lastly, to better understand the dynamics of IPV in emerging adults, additional studies are needed to investigate both victimization and perpetration of IPV to examine not only the bidirectionality, but also the polyvictimization and polyperpetration of these forms of violence [6]. Given that this is the first study to intersect dysfunctional attitudes, sociotropy–autonomy, and the three forms of IPV in emerging adulthood, further replication would be desirable.

### 4.4. Implications

Although this study was exploratory and relatively new, some preventive and clinical implications can be formulated. Even though childhood abuse is an important predictor of IPV, it is difficult to act on it during primary preventions. However, the results underline the work which is required to be continued on the links between beliefs and attitudes which can lead to poorer social skills and, thus, pose problems in romantic conflicts in emerging adulthood. Indeed, negatives beliefs about the self and others are known to underpin sociotropic and autonomous personalities [24,27]. Thus, having a belief or personality that leans too much toward autonomy can cause emerging adults to not be able to develop their sociability, as they are not comfortable in interpersonal relationships and may have difficulty accessing help in case of problems. Conversely, emerging adults who have sociotropic traits may display inappropriate reactions in interpersonal relationships and overreact in the event of conflicts or problems. Working on social skills (e.g., assertiveness or communication) can make it possible to better interact with others, especially in romantic relationships which are important in emerging adulthood. Sociotropy–autonomy could be an indicator worthy of investigating in order to measure social skills before and after IPV prevention programs [36]. On a clinical level, work with cognitive behavioral therapies can be introduced for sociotropic patients on alleviating notions of total abandonment and deprivation of gratification, whereas, for autonomic patients, mastery techniques aimed at invalidating notions of helplessness and incompetence should be actioned [16]. It is all the more important to act on these dysfunctional beliefs, since they directly impact emotions and behaviors [25]. Although these dimensions are principally studied as vulnerability factors for depression, they can also be studied for IPV, given that there are common processes leading to interpersonal problems linked to common psychological vulnerabilities [25,52].

## 5. Conclusions

In summary, the present study expands our knowledge of the relationship between experiences of vulnerability and IPV. The results showed that, after checking for childhood abuse, dysfunctional attitudes, sociotropy, and autonomy were related to IPV victimization for at least one type of violence (i.e., psychological, physical, and sexual) and one scale of severity (minor or severe). Autonomy seemed to be the most associated with the different forms of IPV victimization and its severity. The results did not highlight any clear specificities with respect to the different forms of IPV and their severity. Identifying the factors of vulnerability to IPV victimization could help set up preventive interventions. Our findings are preliminary, and additional research is needed to confirm these links.

## Figures and Tables

**Table 1:** Sex difference in prevalence and frequency of IPV.

	Prevalence					Score of Exposure to IPV		
		Women *n* = 786		Men *n* = 143		Women *n* = 786	Men *n* = 143	
Types of IPV	*n*	%	*n*	%	*χ* ^2^	*M* (SD)		Z
Psychological violence	507	64.5	88	61.5	0.46	14.96 (21.64)	12.78 (16.67)	−0.79
Minor	401	51.0	71	49.7	0.57	13.55 (17.70)	11.48 (13.65)	−0.82
Severe	106	13.5	17	11.9		7.35 (12.13)	7.41 (8.85)	−0.48
Physical violence	130	16.5	26	18.2	0.23	11.88 (29.11)	7.04 (10.66)	−0.37
Minor	95	12.1	21	14.7	0.95	9.15 (18.84)	6.58 (8.63)	−0.20
Severe	35	4.5	5	3.5		11.43 (20.86)	5.00 (6.52)	−0.54
Sexual violence	176	22.4	14	9.8	11.81 **	7.78 (11.12)	5.86 (8.14)	−30.54 ***
Minor	166	21.1	14	9.8	12.25 **	7.43 (9.95)	5.86 (8.14)	−3.51 ***
Severe	10	1.3	0	-		7.00 (5.66)	-	

Note. Prevalence corresponds to have experienced at least one act of IPV in the past year, and score of exposure to IPV corresponds to the frequency of IPV events experienced over the past year. ** *p* < 0.01, *** *p* < 0.001.

**Table 2:** Correlations between severity of IPV and sociodemographic variables.

	1	2	3	4	5	6	7	8	9
1. Minor psychological violence	-								
2. Severe psychological violence	0.39 ***	-							
3. Minor physical violence	0.47 ***	0.40 ***	-						
4. Severe physical violence	0.28 ***	0.33 ***	0.42 ***	-					
5. Minor sexual violence	0.29 ***	0.33 ***	0.22 ***	0.13 ***	-				
6. Severe sexual violence	0.12 ***	0.22 ***	0.14 ***	0.14 ***	0.22 ***	-			
7. Age	0.03	0.03	−0.01	0.01	−0.01	0.00	-		
8. Education level	−0.08 *	−0.03	−0.10 **	−0.08 *	−0.01	−0.03	0.44 ***	-	
9. Childhood abuse	0.15 ***	0.14 ***	0.15 ***	0.04	0.11 *	0.08 *	−0.01	−0.16 ***	-

Note. * *p* < 0.05, ** *p* < 0.01, *** *p* < 0.001.

**Table 3:** Simple correlations and partial correlations among severity of IPV, dysfunctional attitudes, and sociotropy–autonomy, ^a^ controlling for childhood abuse.

	Dysfunctional Attitudes				Sociotropy			Autonomy		
	DAS1	DAS2	DAS3	DAS4	S1	S2	S3	A1	A2	A3
Minor psychological violence	0.08 * (0.05)	0.09 ** (0.07 *)	0.09 ** (0.08 *)	0.05 (0.03)	0.07 * (0.06)	0.06 (0.01)	−0.03 (−0.03)	0.01 (0.02)	−0.02 (−0.04)	0.08 * (0.06)
Severe psychological violence	0.07 * (0.05)	0.11 ** (0.09 **)	0.08 * (0.08 *)	0.08 * (0.07 *)	0.04 (0.02)	−0.02 (0.00)	0.00 (0.00)	0.03 (0.04)	0.10 ** (0.08 *)	0.09 * (0.07 *)
Minor physical violence	0.06 (0.04)	0.08 * (0.05)	0.08 * (0.07 *)	0.08 * (0.05)	0.07 * (0.05)	0.02 (0.04)	−0.02 (0.02)	0.01 (0.02)	0.05 (0.03)	0.06 (0.05)
Severe physical violence	0.02 (0.02)	0.06 (0.05)	0.08 * (0.08 *)	0.01 (0.01)	0.01 (−0.01)	−0.02 (−0.01)	−0.02 (−0.02)	0.06 (0.06)	0.07 (0.06)	0.10 ** (0.09 **)
Minor sexual violence	0.05 (0.04)	0.09 ** (0.08 *)	0.05 (0.04)	0.05 (0.04)	0.08 * (0.07 *)	0.03 (0.04)	0.05 (0.05)	−0.01 (−0.01)	0.06 (0.04)	0.09 ** (0.08 *)
Severe sexual violence	0.05 (0.04)	0.09 ** (0.08 *)	0.06 (0.06)	0.04 (0.04)	0.05 (0.02)	−0.01 (0.00)	−0.01 (−0.01)	−0.01 (−0.02)	0.05 (0.04)	0.03 (0.02)
Childhood abuse	0.12 ***	0.19 ***	0.07 *	0.11 **	0.05	−0.11 **	−0.01	−0.05	0.17 ***	0.12 ***

Note. ^a^ Partial correlations are reported in parentheses. DAS1 = Seeking the esteem of others; DAS2 = The capacity to oppose others; DAS3 = Independence from others; DAS4 = Seeking the approval of others; S1 = Importance given to others; S2 = Worry about separation and looking for support; S3 = Attention to others; A1 = Goal achievement and independence; A2 = Attraction to loneliness; A3 = Valorization of freedom of movement and action. * *p <* 0.05, ** *p <* 0.01, *** *p <* 0.001.

**Table 4:** Results of multinomial logistic regression on severity of IPV, dysfunctional attitudes, and sociotropy–autonomy (*N* = 929).

Type of Violence	Psychological		Physical		Sexual	
Variables	Minor *n* = 472 OR 95 % CI	Severe *n* = 123 OR 95 % CI	Minor *n* = 116 OR 95 % CI	Severe *n* = 40 OR 95 % CI	Minor *n* = 180 OR 95 % CI	Severe *n* = 10 OR 95 % CI
Sex	1.14 [0.77–1.69]	1.39 [0.79–2.53]	0.71 [0.42–1.21]	1.36 [0.50–3.66]	2.71 ** [1.51–4.87]	-
Age	1.06 ** [1.02–1.12]	1.08 * [1.01–1.15]	1.00 [0.94–1.07]	1.04 [0.94–1.14]	1.01 [0.96–1.06]	1.04 [0.86–1.26]
Education level	0.96 [0.90–1.01]	0.95 [0.88–1.04]	0.95 [0.88–1.03]	0.86 ** [0.77–0.96]	1.00 [0.94–1.07]	0.97 [0.75–1.25]
Childhood abuse	1.05 * [1.00–1.10]	1.13 *** [1.06–1.21]	1.14 *** [1.08–1.21]	1.07 [0.97–1.18]	1.06 * [1.01–1.12]	1.18 * [1.01–1.38]
**Dysfunctional attitudes**						
DAS1	1.01 [0.99–1.02]	1.00 [0.98–1.02]	1.00 [0.98–1.02]	0.99 [0.97–1.03]	1.00 [0.99–1.02]	0.98 [0.93–1.03]
DAS2	0.99 [0.96–1.02]	0.99 [0.95–1.04]	0.99 [0.96–1.04]	0.99 [0.92–1.05]	1.00 [0.96–1.03]	1.14 * [1.01–1.29]
DAS3	1.00 [0.96–1.02]	1.04 [0.97–1.11]	1.00 [0.94–1.07]	1.14 ** [1.04–1.27]	1.00 [0.96–1.06]	1.04 [0.85–1.28]
DAS4	0.97 [0.92–1.02]	1.02 [0.95–1.11]	1.01 [0.94–1.09]	0.94 [0.84–1.06]	1.00 [0.94–1.07]	0.90 [0.70–1.15]
**Sociotropy**						
S1	1.01 [0.99–1.04]	1.01 [0.98–1.05]	1.04 * [1.00–1.07]	1.00 [0.95–1.05]	1.00 [0.97–1.03]	1.07 [0.95–1.20]
S2	1.01 [0.99–1.04]	1.01 [0.97–1.05]	1.02 [0.98–1.06]	1.01 [0.95–1.07]	1.01 [0.97–1.03]	0.99 [0.88–1.11]
S3	0.97 [0.94–1.01]	0.97 [0.92–1.03]	0.97 [0.92–1.02]	0.98 [0.90–1.06]	1.01 [0.97–1.06]	0.92 [0.78–1.09]
**Autonomy**						
A1	1.02 [0.99–1.05]	1.02 [0.97–1.06]	1.00 [0.96–1.04]	1.03 [0.96–1.10]	0.98 [0.95–1.02]	0.99 [0.87–1.12]
A2	0.97 * [0.94–0.99]	1.01 [0.96–1.06]	1.02 [0.97–1.07]	1.00 [0.93–1.08]	1.02 [0.98–1.05]	1.08 [0.93–1.26]
A3	1.01 [0.97–1.06]	1.01 [0.97–1.10]	0.97 [0.91–1.03]	1.09 [0.99–1.20]	1.06 * [1.01–1.11]	1.01 [0.82–1.23]

Note. Psychological violence model: Cox and Snell R^2^ = 0.06, Nagelkerke R^2^ = 0.07, *χ*^2^ (28) = 59.48, *p* < 0.001. Physical violence model: Cox and Snell R^2^ = 0.07, Nagelkerke R^2^ = 0.10, *χ*^2^ (28) = 63.56, *p* < 0.001. Sexual violence model: Cox and Snell R^2^ = 0.05, Nagelkerke R^2^ = 0.08, *χ*^2^ (28) = 51.98, *p* < 0.01. The reference group was absence of violence. DAS1 = Seeking the esteem of others; DAS2 = The capacity to oppose others; DAS3 = Independence from others; DAS4 = Seeking the approval of others; S1 = Importance given to others; S2 = Worry about separation and looking for support; S3 = Attention to others; A1 = Goal achievement and independence; A2 = Attraction to loneliness; A3 = Valorization of freedom of movement and action. * *p* < 0.05, ** *p* < 0.01, *** *p* < 0.001.

## Data Availability

The datasets generated and analyzed during the current study are available from the corresponding author on reasonable request.
